# Burch Procedure: A Historical Perspective

**DOI:** 10.1055/s-0042-1744312

**Published:** 2022-02-18

**Authors:** Gisele Vissoci Marquini, Zsuzsanna Ilona Katalin de Jarmy di Bella, Marair Gracio Ferreira Sartori

**Affiliations:** 1 Department of Gynecology, Universidade Federal de São Paulo, São Paulo, SP, Brazil.

**Keywords:** surgical therapy, stress urinary incontinence, surgery, history, burch colposuspension, terapia cirúrgica, incontinência urinária de esforço, cirurgia, história, Burch colposuspension

## Abstract

**Introduction** The Burch procedure (1961) was considered the gold standard treatment for stress urinary incontinence (SUI) before the midurethral slings (MUSs) were introduced, in 2001.

**Objective** This historical perspective of the Burch's timeline can encourage urogynecological surgeons to master the Burch technique as one of the options for surgical treatment of SUI.

**Search Strategy and Selection Criteria** A bibliographic search was performed in the PubMed and National Library of Medicine (NIH) databases with the terms *Burch colposuspension* AND *history* AND *stress urinary incontinence* in the last 20 years. The original article by Burch (1961) was included. The references were read by three authors. The exclusion criterion was studies in non-English languages. Biomedical Library Special Collections were included as historical relevant search.

**Data Collection, Analysis and Main Results** Some modifications of the technique have been made since the Burch procedure was first described. The interest in this technique has been increasing due to the negative publicity associated with vaginal synthetic mesh products. Twenty-nine relevant articles were included in the present review article, and numerous trials have compared Burch colposuspension with MUS.

**Conclusion** This historical perspective enables the scientific community to review a standardized technique for SUI. Burch colposuspension should be considered an appropriate surgical treatment for women with SUI, and an option in urogynecological training programs worldwide.

## Resumo

**Introdução** O procedimento de Burch (1961) foi considerado o tratamento padrão ouro para a incontinência urinária de esforço (IUE) antes da introdução dos slings de uretra média (SUMs), em 2001.

**Objetivo** Esta perspectiva histórica da linha do tempo do procedimento de Burch pode encorajar os cirurgiões uroginecológicos a dominar a técnica deste procedimento como uma das opções para o tratamento cirúrgico da IUE.

**Estratégia de busca e critérios de seleção** A busca bibliográfica foi realizada nas bases de dados PubMed e National Library of Medicine (NIH) com os termos *Burch colposuspension* AND *history* AND *stress urinary incontinence* nos últimos 20 anos. O artigo original de Burch (1961) foi incluído. As referências foram analisadas por três autores com exclusão de estudos em idiomas diferentes do inglês. Coleções de bibliotecas biomédicas foram incluídas por ordem de relevância histórica.

**Coleta de dados, análise e principais resultados** Algumas modificações de técnica foram realizadas desde que o procedimento de Burch foi inicialmente descrito. O interesse por essa técnica vem aumentando devido à publicidade negativa associada aos produtos de tela sintética vaginal. Vinte e nove artigos relevantes foram incluídos, e vários estudos compararam a colposuspensão de Burch com SUMs.

**Conclusão** Essa perspectiva histórica possibilita à comunidade científica revisar uma técnica padronizada para a IUE. A colposuspensão de Burch pode ser considerada um tratamento cirúrgico adequado para mulheres com IUE, e uma opção em programas de treinamento uroginecológico em todo o mundo.

## Introduction

Stress urinary incontinence (SUI) is a prevalent condition that interferes with women's health-related quality of life. It is generally attributable to urethral hypermobility as a result of diminished urethral support, although there can also be a component of urethral sphincter weakness. In women with incontinence secondary to urethral hypermobility, retropubic colposuspension surgery (or urethropexy) is a traditional repair that surgically elevates and reinforces the periurethral tissue. 1

There are several colposuspension techniques, although none is as commonly performed as the Burch procedure. The Burch procedure was first described by Dr John Chistopher Burch in 1961. 2 Burch colposuspension was considered as the gold standard retropubic colposuspension surgical treatment before Ulmsten and Petros 3 presented the tension-free vaginal tape (TVT) procedure in 1995. Consecutively, Delorme 4 practiced the transobturator tape (TOT) (outside-in) procedure in 2001, commonly known as midurethral sling (MUS). Although the colposuspension procedure was once considered the gold standard in the treatment of SUI, its number has waned since the turn of the 21 ^st^ century, following the introduction of the MUS. 1

Notwithstanding, in 2011, the Food and Drug Administration (FDA) issued a notification on the serious complications associated with transvaginal mesh for the surgical treatment for pelvic organ prolapse (POP). 5 6 7 Unfortunately, the negative publicity associated with vaginal synthetic mesh products has extended to MUSs for treatment of SUI. Subsequently, the interest in colposuspension procedures has been rekindled as women and practitioners alike sought alternative surgical treatment options for SUI. As a result, the Burch procedure, a satisfactory correction of almost all types of cystocele by the abdominal approach, continues to have a place in the operative armamentarium of the gynecologists and urologists. 1 2 In addition, Burch colposuspension is still a frequently performed and efective surgical procedure for SUI, especially when there is a need for concomitant pelvic surgery. With the advancements in laparoscopic techniques, laparoscopic Burch colposuspension is gaining popularity as a non-mesh alternative, minimally invasive SUI surgery, which is as effective as open surgery. 8 9 10

Given this scenario, the present historical perspective aims to write a narrative review, a systematic search on the history of the Burch procedure. This review can encourage urogynecological surgeons to master the Burch technique as one of the options for the surgical treatment of SUI.

## Methods

To compose the timeline of Burch's surgery, a bibliographic search was performed in the PubMed and National Library of Medicine (NLM) databases with the terms *Burch colposuspension* AND *history* AND *stress urinary incontinence* in the last 20 years (2001–2021), since the advent of mid-urethral slings. The last date of search was included due to the original Burch's article (Burch, 1961). 2 The references were read by three authors. The exclusion criterion was studies in non-English languages. Biomedical Library Special Collections 11 were included as historical relevant search. Twenty-seven recommended studies were included based on a qualitative or exploratory research strategy in areas in which there is little accumulated and systematized knowledge, with higher level of evidence. The studies included historical articles on biographies and the first publication of the cited technique. Two other studies were included before the study's inclusion date because they were relevant historical publications. 3 12

## Results and Discussion

### Burch's Origins: Dr. John Christopher Burch and his Legacy

John Christopher Burch, eldest son of Dr. Lucius E. Burch and Sarah Polk (Cooper) Burch, was born on July 21, 1900, in Nashville, TN. He attended Vanderbilt University from 1917 to 1919 and then entered medical school, graduating in 1923 as Founder's medalist. After completing residence programs in Boston and New York and studying in Europe, he returned to Nashville in 1926 to begin his practice at the Burch Clinic and to begin his long career of teaching at Vanderbilt Medical School. 11

**Fig. 1.**
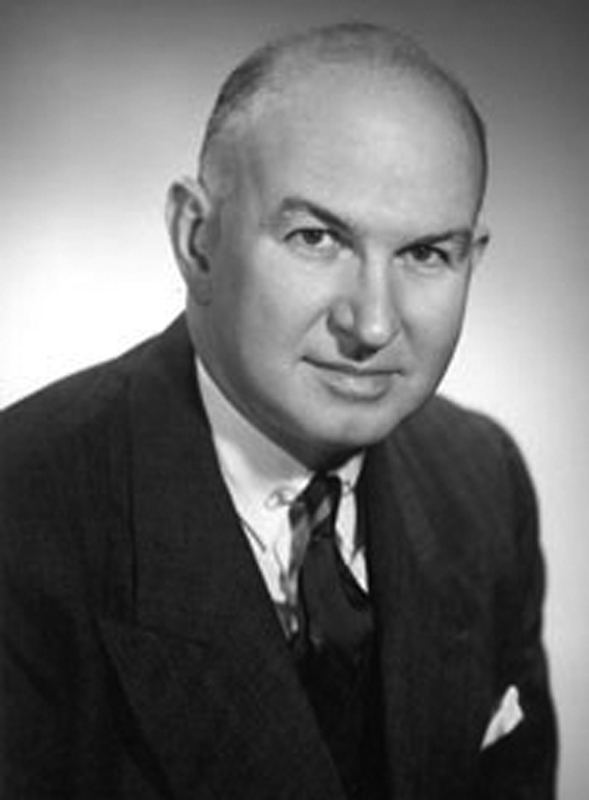
Dr. John C. Burch (1900-1977): professor at Vanderbilt Medical School. He served as the Chair of the department of obstetrics and gynecology. **Source:** Vanderbilt University. 11

Dr. Burch is remembered for his service as Dean of the Vanderbilt Medical School from 1914 to 1925 and chairman of the department of obstetrics and gynecology until 1945. He served as professor not only in the field of urogynecology, but also obstetrics and gynecology. In 1965, as shown in Figure 1 , he became emeritus. 11 13

**Fig. 2.**
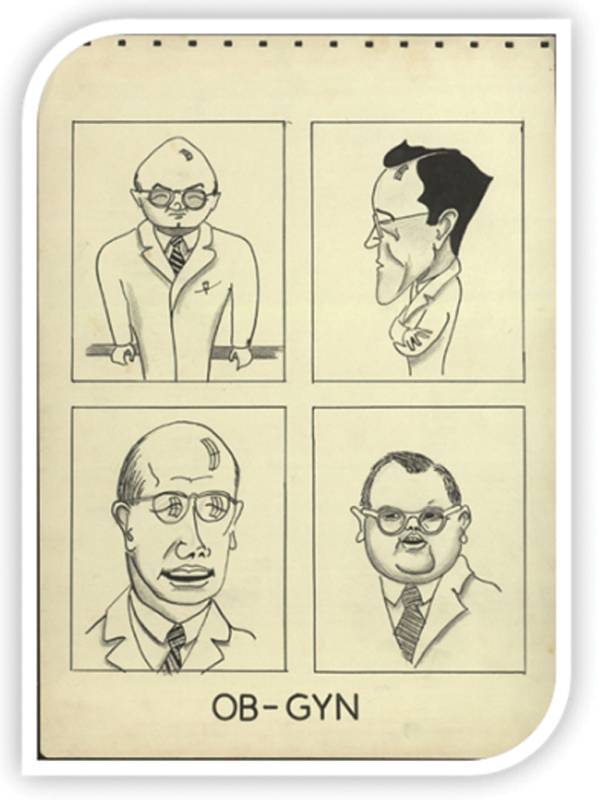
Vanderbilt Medical School. Description: Caricatures of faculty members of the department of obstetrics and gynecology, by medical student Wallace Clyde (1954). Clockwise, from the top left: John Burch, Claiborne Williams, Howard Morgan, Edwin Williams. **Source:** Vanderbilt University. 11

During his career, Dr. Burch authored more than 150 articles. His book *Hysterectomy* is considered a classic. During World War II, he served as chief of the surgical service at Brooke General Hospital in Fort Sam Houston, Texas. Over his long career at Vanderbilt, John Burch taught some 2,000 medical students and trained more than 300 interns and residents. 11 Dr. John C. Burch possessed a rare combination of medical talents. He was a beloved teacher and practitioner and a skilled surgeon and researcher. Burch's kind disposition and proximity to the students were remarkable, as demonstrated in the Figure 2 , in which caricatures of faculty members of the department of obstetrics and gynecology were drawn by a medical student and presented at a departament meeting. 11

**Fig. 3. A -:**
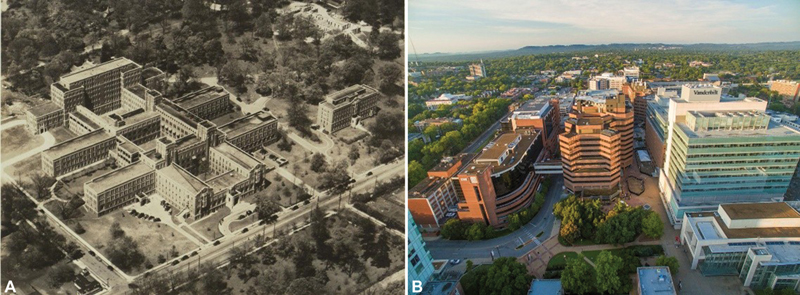
Aerial view of Vanderbilt Medical Center Campus, Nashville, TN, 1938. This aerial view of what is now the Medical Center campus shows the Vanderbilt Hospital and Medical School as it looked after the construction of the D-wing (top left), in 1938. **B -** Aerial view of Vanderbilt Medical Center Campus, Nashville, Tennessee, 2019. **Source:** Vanderbilt University. 11

Currently, The Vanderbilt University is considered one of the top 15 universities in the United States and one of the top 50 in the world. Figures 3A and B show an aerial view of the Vanderbilt Medical Center Campus in 1938 and 2019, respectively.

**Fig. 4.**
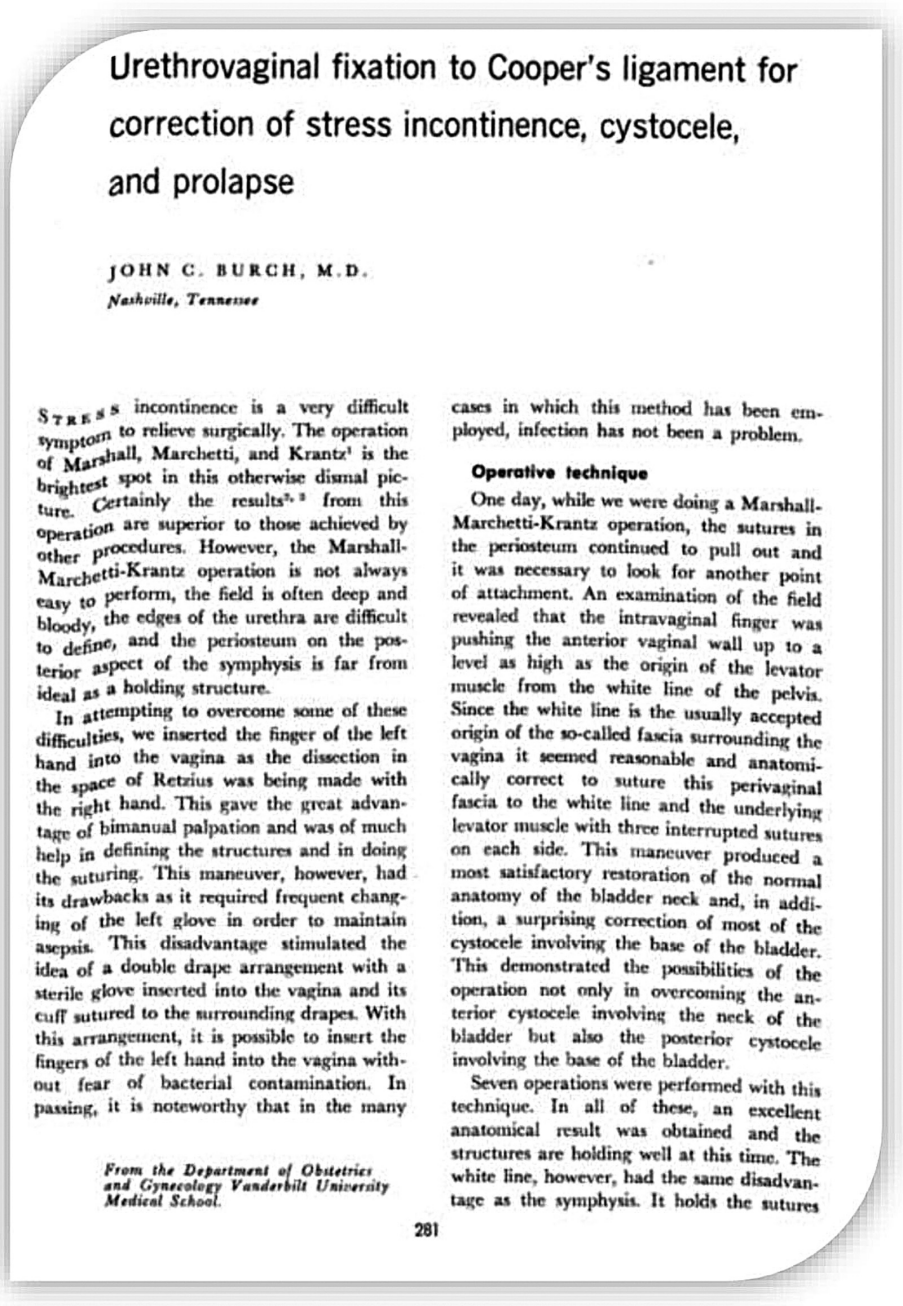
First page of the original article by Burch– 1961. **Source:** Delorme (2001). 4

In 1961, John C. Burch 2 presented a modified colposuspension technique. The first retropubic suspension for the treatment of SUI, also known as Marshall-Marchetti-Krantz (MMK), was described in 1949 by Marshall et al. 14 , with the peri-urethral tissue being sutured to the posterior face of the pubic symphysis. Burch performed the surgical procedure fixing the periurethral/perivesical tissues to the Cooper's ligament. 13 15 In his original article, Burch initially advocated for attaching the paravaginal fascia to the tendinous arch of the fascia pelvis, as shown in Figure 4 . 4 This point of attachment was later changed to the Cooper ligament in order to provide a more secure fixation. The MMK procedure fixes the bladder neck to the periosteum of the symphysis pubis. Historically, the MMK procedure has similar rates of short-term cure as those of the Burch procedure; however, it carries a risk of osteitis pubis (0.7%) that is not present with the Burch technique. 1 16

### Classic Description of Burch Colposuspension

The following steps describe the original Burch procedure (1961). 2

“The dissection to expose Cooper's ligament and the fascia surrounding the vagina begins by breaking through the endoabdominal fascia, which descends from the anterior abdominal wall onto the symphysis and superior ramus of the pubic bone. The most convenient breakthrough site is at the lower angle of the abdominal wound and, when the fascia is broken through, the fingers rest on the bare bone of the symphysis. Now with the left index finger in the vagina and continuing· in this plane, the endoabdominal fascia is stripped from Cooper's ligament and the side wall of the pelvis by the right hand. This plane is relatively avascular and keeping in it avoids the rupture of many small vessels and prevents a most troublesome ooze. In working downward to the fascia surrounding the vagina, the right hand sweeps the endoabdominal fascia from the lateral structures with a lateral and superior motion. When the lateral edge of the vagina becomes apparent, further medial dissection will usually outline to some degree the edge of the bladder. 2

The distinctness of the bladder edge is not always sharp, especially in the obese, and in passing the suture into the perivaginal fascia, one can easily penetrate the bladder. 2

For suture material, number 2 chromic catgut has so far proved satisfactory. Perhaps stainless-steel wire or even fascia may be the eventual choice. The point on the vagina through which the needle and suture have been passed is now matched to Cooper's ligament, and the needle is passed through this ligament and tied by the assistant as the operator pushes up with the intravaginal finger. Three such sutures are passed on each side. The abdomen is closed, the legs retracted and perineorrhaphy and posterior colporrhaphy done if indicated.” 2

The relevant step in the development of the operation was the utilization of the Cooper ligament as a point of fixation. This strong thick band of fibrous tissue runs along the superior surface of the superior ramus of the pubic bone and is ideal from the standpoint of both passing and holding a suture. 2 Burch noticed that this maneuver produced a most satisfactory restoration of the normal anatomy of the bladder neck and, in addition, a surprising correction of most of the cystocele involving the base of the bladder. 2

### Burch's Variations and Outcomes

Variations of the description of the classic Burch technique can be explained in didatic steps 1 9 17 :

1. Either a Pfannenstiel or straight midline subumbilical incision is made (at least 5 cm).
2. The retropubic space is exposed, and the peritoneum is swept superiorly. The periurethral fat is removed for adequate visualization of the anterolateral vaginal wall.
3. A Foley catheter is inserted per the urethra, and the balloon is inflated. With an index finger in the vagina and gentle traction on the catheter, the bladder neck with the Foley balloon is palpable. With an assistant providing exposure by retracting the bladder medially and superiorly, the endopelvic and vaginal fascia are visible.
4. Two (or 3) absorbable stitches are then placed through the endopelvic and vaginal fascial complex, using the index finger to determine the appropriate depth (care should be taken to not violate the vaginal mucosa). The most cephalad suture is usually placed at the level of the bladder neck (2 cm lateral), and sutures are placed about 1 cm apart caudally.
5. The vaginal sutures are then placed through the Cooper ligament and tied loosely (2- to 4-cm suture bridge between the vagina and the Cooper ligament) in a tension-free manner. 1 9 17

According to Burch, 2 his experience with the Cooper ligament urethrovaginal suspension indicates that it is a superior operation for SUI. It achieves a remarkable degree of correction of the deformity of cystocele and provides, for the first time, a satisfactory correction of almost all types of cystocele by the abdominal approach. It can be combined with abdominal hysterectomy and perineorraphy in the treatment of uterine prolapse, but, in these cases, the danger of the subsequent development of enterocele must be recognized, and appropriate precautions must be taken. 2

The critical aspects of the Burch procedure, regardless of surgical approach, are to obtain adequate exposure and to avoid reapproximating tissue under undue tension. The surgical goal is to loosely approximate the Cooper ligament to the periurethral tissue in order to allow postoperative adhesion formation that provides broad support for the urethra and bladder neck. To date, there are no randomized trials to suggest superiority of one suture type over another; however, most surgeons use absorbable suture. In addition, reviews have shown no difference in outcomes whether placing 2, 3, or 4 sutures per side, although, as mentioned above, it has been demonstrated that one suture per side is insufficient. 1 It is also critical to understand that although the Burch colposuspension does suspend the bladder neck and may repair small cystoceles, it is insuficient for repairing significant anterior pelvic organ prolapse (POP). Hence, women with significant prolapse defects with concomitant SUI undergoing colposuspension should additionally have a dedicated cystocele repair. 1 9

Over the years, several authors and surgeons have presented numerous modifications of the original operation described by Burch. 17 The procedure was further modified by Tanagho, 12 in 1976, to its current state, in which the paravaginal sutures are placed further laterally from the urethra, and a looser approximation of tissues is undertaken. Over time, Burch colposuspension has been adapted for laparoscopy and modifications of the original technique, such as synthetic mesh use to secure paraurethral support, have been introduced. 17

The wealth of data from comparative and observational studies assessing the outcome of Burch colposuspension has been reported in numerous textbooks and structured summary publications. 17 18 Since it first description in 1961, there has been a multitude of randomized controlled trials including Burch colposuspension. Fifty-five trials involving a total of 5,417 women have been included in the current Cochrane review about open retropubic colposuspension. 9

Numerous trials have compared Burch colposuspension with MUS. Several observational and randomized studies have showed similar eficacy and lower morbidity for MUS procedures compared to Burch colposuspension. 8 9 10 17 Others have cited that there was no statistically significant difference in subjective cure rates, but the objective cure rates tended to be higher for MUS. 19 20 21 22 Even if the definition of objective cure varied widely between available studies, many of them still report that MUSs (retropubic slings or transobturator slings) are superior to Burch colposuspension surgery. 10 17 23 24 According to those outcomes, patients receiving midurethral tapes have significantly higher overall and objective cure rates than those receiving Burch colposuspension. In addition, in a survey among professionals, Burch colposuspension would have been chosen only by a minority of surgeons. 23 24 25

In general, bladder injury, voiding dysfunction, and hematoma can be reported equally with Burch colposuspension or midurethral tapes. Midurethral slings may exhibit a much higher risk of intraoperative complications, such as bladder perforation and urinary retention, than the Burch procedure. 23 24 On the other side, the MUS placement was associated with shorter operating time, length of hospitalization, and time for resuming normal activity. 19 20 21 22 However, even if the length of hospital stay may be longer for Burch colposuspension, with this technique, there is no possibility of mesh extrusion as a complication. 23 24

Basically, data on long-term effectiveness and adverse events are, however, limited, especially around the comparative adverse events profiles of MUS and non-MUS procedures. A better understanding of complications after surgery for SUI is imperative. 24

Although mesh slings remain a strong option to surgical treatment for SUI, there has been renewed interest in autologous fascial slings (AFSs) for the treatment of SUI, because of the investigations of mesh safety for POP. 26 27 The fascial sling is an effective operative technique in patients who have undergone previous operations for incontinence and a second non-synthetic mesh option for SUI. A retrospective study, with a robust sample of women (463 patients), evaluated whether the conventional AFS was superior or equal to the readjustable transobturator sling in efficacy and safety in women with SUI, and it showed that both techniques had similar subjective efficacy rates. However, the transobturator sling demonstrated fewer postoperative surgical complications when compared with the AFS, such as morbidity of wound infection or hematoma. 26

Currently, regulations surrounding the use of mesh implants for POP differ depending on the country and can influence the number of procedures of synthetic slings for SUI. Although in the United States of America (USA), the United Kingdom (UK), Canada, Australia, New Zealand, and France, transvaginal mesh implants for POP have been removed from the market, in most mainland European countries, Asia, and South America, they are still available as a surgical option for POP correction. 28

Then, it is expected that in the USA, the UK, Australia, New Zealand, Canada, and France, with the removal or the restriction of transvaginal mesh implants as a surgical option for POP, the spectrum of urogynecological operations, including surgeries for SUI, might be greatly affected. Conventional transvaginal native tissue repair and abdominal (open, laparoscopic) surgical procedures, as well as Burch colposuspension or non-synthetic mesh procedures are expected to be increasingly performed. This requires appropriate training of younger physicians. 28

According to this scenario, a strong point in the study is the heterogeneous source of historical research current overview of Burch's surgery. Encouraging the study of this established technique can rescue an effective surgical option for SUI in a setting of valorization of individualized conducts.

A limitation of the present study is the non-systematic methodology. The search for a historical description of Burch's surgery overlooked classic articles without a systematic review. However, these documents deserve to be part of the knowledge of the urogynecological surgeon due to the scientific contribution in the development of surgical techniques throughout the century in urogynecology.

The authors believe that the limitation of the non-systematized methodology does not detract from the study because the objective was to demonstrate, through a collection of its history, a surgical treatment option for urinary incontinence. A technique that has survived for more than 50 years, still recommended today, carries its scientific value.

The Burch colposuspension has a 50-plus year history demonstrating strong long-term outcomes with minimal complications. Iterations of the procedure, including laparoscopic, robotic, and mini-incisional approaches, appear to have equal efficacy to the open procedure. Although the current use of the Burch colposuspension has waned with the growing shift toward sling surgery, it continues to have a role in the treatment of SUI.

Specifically, given satisfactory long-term outcomes over the course of the last half-century, it seems that the Burch colposuspension should be considered an appropriate surgical treatment for any woman with SUI, especially in settings where vaginal access is limited, where intra-abdominal surgery is already planned, or if mesh is contraindicated. 1 9 17 29

On the other side, the National Institute for Care and Health Excellence (NICE) guidelines include amongst their recommendations that laparoscopic Burch colposuspension should not be used as a routine procedure for the treatment of SUI in women. It was highlighted, that the procedure should be performed only by surgeons with appropriate training as well as expertise working in a multidisciplinary team, and women should be advised about the limited evidence. 29

## Conclusion

Finally, the Burch procedure has an ongoing role in the surgical repair of female SUI and should remain in the surgical repertoire of female pelvic medicine and reconstructive surgeons. The authors suggest new long-term follow-up studies for the association of the Burch colposuspension technique in the setting of laparoscopic, robotic, or minimally invasive urogynecological surgery. The science has no answer, so far, whether urogynecology has started a journey back to the future by revitalizing the Burch procedure. However, in light of such a development, training in both open and laparoscopic Burch colposuspension should nowadays be provided in urogynecological fellowship and training programs worldwide.
